# Open-label trial of three dosage regimens of fixed-dose combination of artemisinin and naphthoquine for treating uncomplicated *falciparum* malaria in calabar, Nigeria

**DOI:** 10.1186/1475-2875-11-413

**Published:** 2012-12-11

**Authors:** Martin M Meremikwu, Friday Odey, Chioma Oringanje, Angela Oyo-ita, Emmanuel Effa, Ekpereonne B Esu, Eyam Eyam, Olabisi Oduwole, Vivian Asiegbu, Ambrose Alaribe, Emmanuel N Ezedinachi

**Affiliations:** 1 Institute of Tropical Diseases Research and Prevention, University of Calabar Teaching Hospital (UCTH), Calabar, Cross River State, Nigeria; 2 Department of Paediatrics, University of Calabar, Calabar, Nigeria; 3 Department of Community Medicine, University of Calabar, Calabar, Nigeria; 4 Department of Internal Medicine, University of Calabar, Calabar, Nigeria; 5 Department of Public Health, University of Calabar, Calabar, Nigeria; 6 Department of Medical Laboratory Science, University of Calabar, Calabar, Nigeria

**Keywords:** Falciparum malaria, Artemisinin, Naphthoquine, Combination therapy

## Abstract

**Background:**

The use of anti-malarial drug combinations with artemisinin, or with one of its derivatives, is now widely recommended to overcome drug resistance in falciparum malaria. Fixed-dose combination of artemisinin and naphthoquine is a new generation artemisinin combination therapy (ACT) offered as a single dose therapy. The aim of the study was to assess the therapeutic efficacy, safety and tolerability of three dosage schedules of fixed-dose combination of artemisinin (125 mg) and naphthoquine (50 mg) for treating uncomplicated *Plasmodium falciparum* malaria among adolescents and adults in Calabar, South-east Nigeria.

**Method:**

A total of 121 patients aged ≥15 years with uncomplicated *P*. *falciparum* malaria were enrolled and randomly assigned to three dosage schedules: (A) 700 mg (four tablets) single dose; (B) 700 mg 12-hourly x two doses; and (C) 1,400 mg (eight tablets) single dose. Patients were observed for 28 days, with clinical, parasitological, and haematological assessments.

**Results:**

A total of 108 patients completed the study. The overall 28-day cure rate was 88.9%. Day 28-cure rates of the three dosage schedules were 85.3%, 93.1% and 88.9% for Group A, B and C respectively. Adverse events were few and mild, the commonest being weakness and headache; there was no serious adverse event.

**Conclusion:**

Concerns for emergence of parasite resistance due to the use of artemisinin-naphthoquine as single dose regimen is likely to compromise the usefulness of this potentially important combination treatment. A robust multi-centre trial is recommended to evaluate a three-day regimen with potentials to achieve high cure rates while minimizing the risk of emergence of resistant parasite strains.

## Background

Malaria remains a major public health problem in Nigeria accounting for as much as 30% childhood deaths and 11% maternal mortality in Nigeria [1]. High levels of *Plasmodium falciparum* resistance to chloroquine [2] and sulphadoxine-pyrimethamine [3] preparations led to the worsening of the malaria situation in the country and prompted drug therapeutic efficacy tests (DTET) on artemisinin-based combinations in the country. The results of the DTET informed the change in the malaria treatment policy in 2005 [4].

Nigeria adopted artemisinin based combination therapy (ACT) as the treatment of choice for uncomplicated plasmodium falciparum malaria. The goal of combination therapy is to increase effectiveness of available antimalarial drugs and delay the emergence and spread of drug resistance [5,6]. The strategy is supported empirically by the success of combination therapy in treating tuberculosis and human immunodeficiency virus infections, and by mathematical models [7]. By using two or more drugs with independent mechanisms of action, it is believed that mutations that confer resistance to each drug will only rarely co-exist in the same parasite, thereby minimizing the incidence of resistant strains of the parasite. ACT is, therefore, expected to improve treatment cure rate and delay the emergence of drug resistance [8].

A fixed dose combination of artemisinin-naphthoquine (Arco™) developed by Kunming Pharmaceuticals Corporation (KPC), China was tested in this study. The drug is administered orally as a single dose treatment for uncomplicated falciparum malaria. In addition to the high cure rate of 97% recorded in two earlier reports by Chinese investigators [9,10], this treatment regimen is likely to have the advantage of a high rate of patient compliance given the simple dosage schedule. Naphthoquine is rapidly absorbed reaching peak plasma concentration 2-4 hours after administration [11]. It is slow in action while artemisinin is rapid in action thus artemisinin immediately starts the antimalarial action by rapidly killing all the malaria-causing Plasmodium parasites in the body (about 97% of the parasites in 24 hours) while naphthoquine which stays longer (half life 41–57 hours) starts its own anti-malarial action late, but continues its action long after the elimination of artemisinin [12].

The aim of this study was to assess the therapeutic efficacy, safety and tolerability of fixed dose combination of artemisinin-naphthoquine for the treatment of uncomplicated falciparum malaria among adolescents and adults in Nigeria. Separate trials in younger children (< 5 years and 5–14 years) are planned for the same study area.

## Methods

### Study area

The study was conducted from 6^th^ March 2006 to 19^th^ August 2006 in a Primary Health Centre (PHC) in Ikot Ansa, Calabar of Cross River State. The health centre is located in a semi-urban community with holoendemic *P*. *falciparum* transmission. The level of *Plasmodium falciparum* resistance to chloroquine and sulphadoxine-pyrimethamine is high [1-3].

### Study design and patients enrolment

The study was an open-label, non-comparative therapeutic efficacy clinical trial carried out following the standard WHO protocol for in vivo efficacy [13]. Patients attending out-patient clinic at study site with symptoms suggestive of malaria were screened for the following inclusion criteria: (1) mono infection of uncomplicated *P*. *falciparum* malaria with a diagnosis confirmed by a positive blood smear with asexual forms of *P*. *falciparum*; (2) residence in the study area throughout duration of follow up; (3) age ≥15 years; (4) history of fever within the past 24 hours or axillary temperature ≥37.5°C; (5) parasite density of 500–200,000 asexual parasites/μl; (6) absence of signs of severe malaria as defined by World Health Organization’s criteria [14]) or any other life-threatening condition; and (7) signed informed consent (by patient or from parents/guardian if less than 18 years). Exclusion criteria were: (1) pregnancy; (2) history of allergy to any of the drug components; (3) severe malnutrition; (4) severe anaemia (haematocrit < 15%); (5) history of having taken an anti-malarial in the past two weeks and (6) presence of any other severe illness or any “danger sign” such as inability to sit, stand up, drink, persistent vomiting, convulsions, lethargy or unconsciousness.

### Sample size calculation

The sample size was determined based on the WHO standard protocol for non-comparative therapeutic efficacy studies [13]. Assuming an anticipated population proportion of clinical failure (p) of 10%, confidence level of 95% and study precision (*d*) of 10% point, the calculated sample size for each arm was 35. An adjustment of 20% for follow-up losses and withdrawals was made as recommended in the WHO protocol for studies with follow-up periods > 14 days. The adjusted sample size per arm was 42 participants. The aim was to enrol a minimum of 42 patients per arm or retain at least 35 at completion of follow-up.

### Ethical considerations

The study was approved by the national regulatory authority in Nigeria. Ethical approval was received from the Ethics Committee of University of Calabar Teaching Hospital prior to onset of study. A written informed consent was obtained from each patient (or the parent/legal guardians if < 18 years) prior to enrolment.

### Treatment

Patients were randomly assigned to one of three dosage scheduled of fixed-dose combination of artemisinin-naphthoquine as follows: Group A received four tablets as a single dose; Group B received four tablets twice at 12 hours interval (total of eight tablets); Group C received eight tablets as a single dose. The allocation sequence was generated by simple balloting and concealed in serially numbered, opaque, sealed envelopes. This was opened by the study nurse at the point of drug administration following completion of consent and all clinical procedures. All the treatments were given in the health facility under direct observation of the study personnel. Drugs were given with water and after patient had a meal. Tepid sponging, exposure and administration of paracetamol were used to reduce high fever. Patients were observed for about 30 minutes after administration of drug and treatment was repeated once if patient vomited within 30 minutes of drug administration. Patients that vomited a second time were excluded from the trial and treated as severe illness. All patients were treated as outpatient.

### Clinical and laboratory assessment

Clinical examination including axillary temperature measurement, body weight and height were recorded on day of enrolment (day 0). During each follow-up visit, clinical examination including temperature was performed. Parasitological examination (thin and thick blood films) were performed on enrolment using Giemsa and performed on each follow up visits. Parasite density was enumerated using thick film as described by Shute [15]. Blood films were considered negative if no parasites were seen in 100 oil-immersion fields in a thick blood film. Routine biochemical and haematological laboratory measurements were done on a fraction (25%) of the enrolled patients.

### Follow-up

Follow-up visits were scheduled on days 1, 2, 3, 7, 14, 21 and 28. Packed cell volume (PCV) was estimated on day zero and repeated on days 14 and 28 for all patients using blood sample collected in a heparinized capillary tube, spun for 5 minutes at 5,000 rpm with micro-haematocrit centrifuge and read with micro-haematocrit reader. Participants that did not return on schedule for follow up were visited at home the same day by study field staff.

### Outcome measures

Outcomes were defined based on the WHO guidelines for assessing therapeutic efficacy [13]. The 28-day cure rate i.e. Adequate Clinical and Parasitological Response (ACPR) was the absence of parasitaemia on day 28 without previously meeting criteria for ETF, LCF or LPF was taken as the primary efficacy endpoint. Secondary outcome measures were Early Treatment Failure (ETF – danger signs or complicated malaria or failure to adequately respond to therapy days 0–3 or parasitaemia on day 2 higher than day 0; or parasitaemia on dya 3 ≥ 25% of day 0 values); Late Clinical Failure (LCF – development of danger signs or severe malaria and parasitaemia after day 3 or presence of parasitaemia and fever on days 4 – 28 without previously meeting criteria for ETF); Late Parasitological Failure (LPF – asymptomatic parasitaemia on day 28 without previously meeting criteria for ETF or LCF); the change in the mean packed cell volume from day 0 to day 28, fever and parasite clearance time, safety and tolerability of the treatment regimen. Safety and tolerability assessment consisted of monitoring and recording of all adverse events (AEs – an unfavourable or unintended signs or symptoms or illness that develops or worsens during the period of observation in the study) and Serious Adverse Events (SAEs). SAE was defined as any untoward event that results in death, is life threatening, requires inpatient hospitalization or results in persistent or significant disability/incapacity.

### Quality control

Clinical and laboratory procedures were subjected to internal quality control. There was no external monitor for this study. However, the trial was audited by officials from the regulatory body in Nigeria; the National Agency for Food and Drug Administration and Control (NAFDAC). Standard operating procedures based on the principles of good clinical practice were prepared for measurement of height, weight, temperature, collection of blood samples and calculation of parasite counts. Malaria parasites were counted by two microscopists independently and the average count was taken as the final count. Where there was a marked difference in count (>25%) between study team microscopists, such slides were sent to an external field microscopist to confirm the count. The external microscopist also randomly selected and reviewed 10% of all the slides read by study team microscopists. Haematology and biochemical tests were conducted in the laboratories of the University of Calabar Teaching Hospital with quality control measures in place.

### Statistical analysis

Data generated were recorded in a log book, and individual patients’ case report files were double – entered and analysed first in EPI Info Version 6 and later using SPSS (version 11.0.1). Arithmetic and geometric means as well as standard deviation were calculated for baseline characteristics. One-way ANOVA was used to test for statistical significance and p-values less than 0.05 were considered to be statistically significant. Both intention-to-treat and per protocol analyses were presented for the main outcomes.

## Results

### General characteristics and flow of trial participants

**Figure 1:**
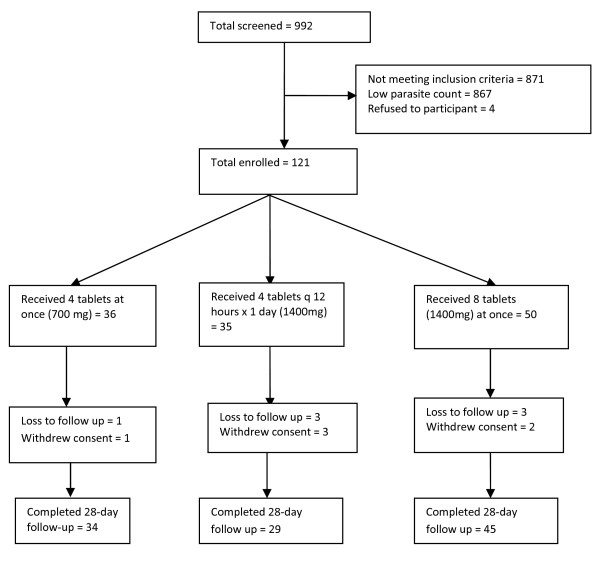
Flow diagram of trial participants.

A total of 992 persons were screened; 348 (35.1%) were males and 644 (64.9%) females. One hundred and twenty-one (121) patients with uncomplicated malaria who fulfilled the inclusion criteria were enrolled: 36 to Group A; 35 to Group B and 50 to Group C (Figure 1).

The commonest reasons for exclusion were low parasite density and recent treatment with anti-malarials. Study participants had similar characteristics at baseline (Table 1).

**Table 1:** Enrolment and baseline data of trial participants

**Participants characteristics**	**Group A**	**Group B**	**Group C**	*p-value
Number enrolled	36	35	50	
Female	27	19	28	
Male	9	16	22	
Mean Age in years (±SD)	29.2	32.8	31.6	0.554
	(±12.4)	(±14.3)	(±13.2)	
Mean Temperature in degrees°C (±SD)	36.6	37.1	37.0	0.439
	(±0.88)	(±0.94)	(±0.96)	
Mean Weight in Kg (±SD)	60.4	65.5	65.2	0.272
	(±11.5)	(±15.3)	(±11.6)	
Mean Height in Cm (±SD)	157.8	160.5	163.3	0.108
	(±18.4)	(±7.8)	(±7.3)	
Mean Day 0 Haematocrit (±SD)	36.9	38.5	39.3	0.104
	(±3.6)	(±5.1)	(±4.8)	
Geomean Day 0 parasite density/uL (95% CI)	1273	1160	1624	0.514
	(924. - 1754)	(842. - 1599.)	(1179–2238)	

*ANOVA.

### Treatment outcome

Table 2 summarizes the treatment outcome for the primary and secondary efficacy endpoints. The overall 28-day cure rate (not confirmed by PCR) was 88.9%. The cure rate for the different treatment groups were 85.3%, 93.1% and 88.9% for Group A, Group B and Group C respectively. A total of 12 (9.9%) patients had late parasitological failure (LPF): 5 in Group A and C respectively and 1 in Group B. There was no early treatment failure (ETF). Table 2 also shows that seven patients (5.8%) were lost to follow up and while six persons (4.9%) withdrew from the study with overall attrition rate of 10.7%.

**Table 2:** Outcome of treating uncomplicated malaria with fixed-dose Artemisinin-Napthoquine combination

Treatment Outcome*	**Group A (%)**	**Group B(%)**	**Group C (%)**
Number enrolled	36	35	50
Number evaluable	34	29	45
28-Day Cure (ACPR) **	29 (85.3%)	27 (93.1%)	40 (88.9%)
(95% conf. Interval)	68.9 – 95.0	77.2 – 99.2	75.9 – 96.3
Late Parasitological Failure	5 (14.7%)	2 (6.9%)	5 (11.1%)
Lost to follow-up	1	3	3
Withdrawn	1	3	2

*ANOVA showed no significant difference in outcome between treatment arms (p = 0.880).

**ACPR = Adequate Clinical and Parasitological Response; with no PCR correction.

### Parasite and fever clearance

**Figure 2:**
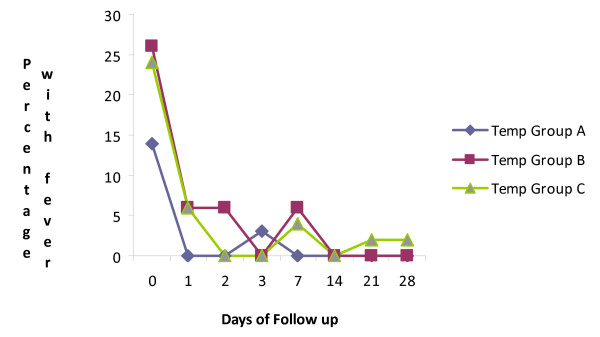
Percentage of patients with fever (temperature ≥37.5°C) following treatment with a fixed combination of Artemisinin- Naphthoquine.

**Figure 3:**
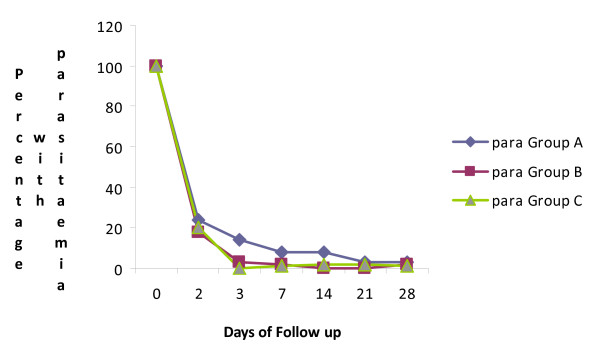
Percentage of patients with parasitaemia following treatment with a fixed combination of Artemisinin- Naphthoquine.

Mean fever clearance time was 24.7 hours (Figure 2). Mean parasite clearance time in this study was 44.8 hours for all study arms. Parasite level dropped by 60% by day 2 and was no longer detectable by day 3 as shown in Figure 3.

### Adverse events

This combination of Artemisinin-Naphthoquine was found to be quite safe. Of the 121 patients that were enrolled, none had serious adverse event (SAE). Table 3 shows the list of adverse events observed as it applies to different treatment arms. Weakness and headache were the most common adverse events reported and were reported more in the 1400 mg single dose group.

**Table 3:** Adverse events among patients treated with Artemisinin-Naphthoquine

**Adverse events**	**Frequency (%)**		
	**Group A**	**Group B**	**Group C**
	**N = 36**	**N = 35**	**N = 50**
Weakness	4 (11.1)	1 (2.86)	7 (14.0)
Itching	1 (2.77)	3 (8.57)	0
Joint pains	1 (2.77)	0	1 (2.0)
Tingling sensation in the head	1 (2.77)	0	0
Headache	3 (8.33)	3 (8.57)	10 (20.0)
Body pains	0	1 (2.86)	1 (2.0)
Catarrh	0	1 (2.86)	2 (4.0)
Nausea	0	2 (5.71)	0
Vomiting	0	1 (2.86)	0
Swollen fingers and feet	0	1 (2.86)	0
Bitter taste	1 (2.77)	1 (2.86)	2 (4.0)
Palpitation	0	1 (2.86)	0
Cold	0	1 (2.86)	0
Hearing impairment	0	1 (2.86)	0
Dizziness	1 (2.77)	1 (2.86)	2 (4.0)
Blisters on lips	0	1 (2.86)	0
Loss of appetite	0	0	1 (2.0)
Drowsy	1 (2.77)	0	0
Cough	0	1 (2.86)	1 (2.0)
Throat itch	0	0	1 (2.0)
Heaviness on stomach	0	0	1 (2.0)
Throat pain	0	1 (2.86)	0
Diarrhoea	0	1 (2.86)	0
Waist pain	0	1 (2.86)	0

### Tolerability

The combination was well tolerated by the study participants. Biochemistry and haematological parameters did not deviate significantly from normal values (data not shown).

## Discussion

This study showed an overall 28-day cure rate of 79.3% for the fixed dose combination of artemisinin-naphthoquine. It was not possible to perform polymerase chain reaction (PCR) to confirm the genetic characteristics of the species reported as treatment failures in this study to determine whether these were due to recrudescence or actually new infections. This cure rate is lower than those reported in other efficacy studies on the same drug, most of which were between 97% and 98% [9-11,16]. Treatment failures in all these studies were confirmed with PCR thereby excluding new infections. It is possible that the failure to exclude possible new infections in this Nigerian study with molecular techniques has contributed to the lower cure rate reported.

Single dose artemisinin-naphthoquine has also been shown to have an efficacy rate comparable to that of artemether-lumefantrine [17]. This study did not compare artemisinin- naphthoquine with another ACT, but an earlier study in the same locality showed comparable cure rates for artemether-lumefantrine (87.0%) and artesunate/amodiaquine (82.5%) [18]. The rapid decrease in the number of persons with parasitaemia by Day 2 of follow-up is indicative of the high therapeutic efficacy characteristic of other forms of ACT already in use. An adequately powered comparative trial of established formulations of ACT with this agent will be required to establish non-inferiority or otherwise, as well as tolerability and adherence to treatment.

The recommended artemisinin-naphthoquine dose for those aged 15 years and above is a single blister (1,000/400 mg) comprising eight tablets (125/50 mg). The exploratory dose-finding assessment performed in this study showed that the treatment arm that received the total recommended dose in two split doses of four tablets (500/200 mg) per dose had a higher 28-day cure rate than those that received the recommended single dose regimen without any difference in tolerability. This observation is limited by the lack of pharmacokinetics data to adequately investigate bioequivalence.

The unique advantage of artemisinin-naphthoquine is its simple dosage schedule, a characteristic which is more likely to be associated with better patient adherence to treatment than ACT regimens that required multiple dosing schedules [19,20]. The second drug in this artemisinin combination therapeutic regimen, naphthoquine has a long elimination half-life and has not been used widely in this environment as a single agent for the treatment of *P*. *falciparum* malaria and, therefore, is able to sustain and complete elimination long after the artemisinin component has waned to below therapeutic levels [10]. While the use of this combination regimen on naive parasite populations may achieve high cure rates, there is however significant risk that widespread single dose use of naphthoquine in this combination regimen could create sufficient pressure on the parasites leading to emergence of increasingly less susceptible mutants and ultimately varying degrees of parasite resistance [14,21]. This raises significant concern about the continued use of this drug combination as a single dose regimen among populations resident in areas with intense perennial transmission where there is likely to be a rapid increase in the pool of people with mutant species of *P*. *falciparum*. Recent evidence from clinical evaluation of a large cohort of children treated with artemether-lumefantrine and artesunate-amodiaquine showed that these artemisinin-based combination treatments are still highly efficacious in Nigeria [22]. However, reports of emergence of strains of *P falciparum* resistant to the artemisinin compound in south-east Asia calls for greater attention to rationale use of ACT regimens [23].

In order to prevent or delay the emergence and spread of *P*. *falciparum* resistance to the component drugs of this potentially beneficial combination regimen (arteminisin-naphthoquine), it would be advisable to use a once daily regimen for a period long enough to ensure complete elimination of all susceptible parasites in each treatment course. In this study, the use of a single-dose regimen of half the recommended dose was explored and revealed a fairly good 28-day cure rate but lower than the rate obtained with the recommended dose. It is likely that administration of single daily dose of 500/200 mg for three days will achieve high cure rates with much lower risk of recrudescence than would be the case with the current practice of single dose of 1,000/400 mg give only for one day. A well designed trial to test this dosage option would be of immense public health importance to avert the likely deterioration of this combination regimen from widespread use as single dose regimen.

One of the key elements in any drug development and evaluation is the issue of safety of the population for which the drug is intended. Artemisinin-naphthoquine was well tolerated and no serious adverse event was recorded in the study. The commonest adverse events were weakness and headache which were reported more in the group that took the recommended dosage. Since both symptoms could also be caused by malaria, it is not certain whether these symptoms were due to the drug alone or caused by the illness itself. This is in keeping with findings from earlier studies in China that showed that this drug is safe and well tolerated [9,10,16]. Larger studies are needed to define the safety and efficacy in different population including children and pregnant women.

## Conclusion

The artemisinin-naphthoquine combination is a potentially important ACT, which deserves further clinical evaluation. While the single dose regimen currently recommended by the developers has a promise for enhanced patient adherence to treatment, it raises serious concerns about the likely emergence of resistant parasite strains to the component drugs. The use of the formulations in reduced strength over a three-day period to minimize the risk of emergence of resistant mutants of *P*. *falciparum* is recommended. A well-designed, adequately powered multicentre randomized controlled trial that meets all GCP requirements will be required to determine how this potentially important combination can be put to the best public health use.

## Competing interests

The authors declare that they have no competing interests.

## Authors’ contributions

ENE and MMM contributed to design of the study; FO, CO and MMM coordinated the study, performed data analysis and drafted the paper; EE, FO and EE performed clinical assessment; CO, OO and VA performed laboratory tests, AA supervised laboratory team, EBE contributed to data analysis and drafting of paper. All authors read and approved the final manuscript.
